# A User-Centered Service Model for Accelerating COVID-19 Diagnostic Innovation: The RADx-rad Dx Core Approach

**DOI:** 10.1109/OJEMB.2025.3568203

**Published:** 2025-05-08

**Authors:** Melissa Ledgerwood-Lee, Alexandra Hubenko, Partha Ray, Yves Theriault, Howard Brickner, Lidia F. Vazquez, Robert Schooley, Aaron Carlin, Alex Clark, Aaron Garretson, Eliah Aronoff-Spencer

**Affiliations:** Qualcomm InstituteUC San Diego 584042 La Jolla CA 92093 USA; Department of MedicineUniversity of California San Diego 8784 San Diego CA 92130 USA; UC San Diego Health, Department of MedicineDivision of Infectious Diseases 278451 La Jolla CA 92093 USA

## I. Introduction

At The end of 2019, the novel SARS-CoV-2 virus emerged in humans, spreading rapidly and leading to the COVID-19 pandemic. The outbreak caused significant morbidity and mortality, prompting governments worldwide to implement lockdowns and masking measures, which resulted in substantial social and economic disruptions. One of the most critical challenges in controlling the virus initially was the lack of diagnostic tests [1], [2], [3]. Effective diagnostic testing is essential for detecting outbreaks and mitigating transmission by allowing for early identification and intervention [4], [5], [6], [7].

However, developing and scaling diagnostic capabilities is inherently complex and requires significant expertise and resources [8], [9], [10], [11], [12]. The process involves multiple stages, including biomarker identification, reagent development, test design, validation, usability and human factors considerations, regulatory compliance, manufacturing, logistics, and commercialization [13], [14], [15], [16], [17], [18]. Each stage presents potential failure points that can hinder a diagnostic tool from successfully reaching the market and being adopted by end-users [19], [20], [21], [22], [23].

In April 2020, the National Institutes of Health (NIH) initiated the RADx-rad initiative to expedite the development of innovative diagnostic technologies for detecting and monitoring SARS-CoV-2 [24], [25]. As part of this program, the Diagnostics (Dx) Core was created to provide essential resources and guidance to RADx-rad grant awardees, supporting them throughout the diagnostic innovation process.

**The Dx Core had two main objectives:**

1) Harmonize data among awardee groups to ensure accurate test development and standardized reporting to the NIH.
2) Offer extensive support and resources to enhance the diagnostic test development process.

To effectively meet these objectives, the Dx Core implemented a user-centered service model that prioritized the needs and outcomes of the users: the grant awardees [26], [27], [28]. This paper outlines the development, implementation, and results of the Dx Core's user-centered approach from December 2020 to December 2024. By establishing this framework, we provide insights into how diagnostic development was supported during the pandemic and present a scalable model that can serve as a blueprint for future diagnostic innovation efforts.

## II. Development of a User-Centered Service Model

### A. Understanding the Users: The RADx-rad Awardees

The first step in developing our service model was understanding our users. Throughout the program, we sent periodic surveys to gather information about the users, their technology, their development progress, and their desired services.

The Dx Core supported 49 awardee groups, with 39 responding to the initial survey. Table I provides a summary of the awardee characteristics, highlighting key details about their diagnostic test characteristics, testing methods, and Technology Readiness Level (TRL), offering insight into the diversity and developmental stages of their diagnostic innovations.

**TABLE I:** RADx-rad Awardee Characteristics. This Table Provides an Overview of the RADx-rad Awardees, Including Their Group Affiliations, Institutional Background (Academic or Private Company), Diagnostic Test Characteristics, Technology Readiness Level (TRL), and the Testing Methods Utilized

Awardee Group Affiliation (N=49)	Count	Tech Readiness Level (N=39)	Count
Novel Biosensing	11	TRL 1: Review of Scientific Knowledge Base	2
PreVAIL kids	8	TRL 2: Development of Product Hypothesis	0
Wastewater	7	TRL 3: Identification of Product Candidate	6
Auto detection & tracing	6	TRL 4: Optimization	12
Chemosensory	4	TRL 5: Initiation of Manufacturing	7
Exosome	4	TRL 6: Production, Regulatory and Clinical Data Submission	2
Multimodal surveillance	4	TRL 7: Scale-up and Phase 2 Clinical Trials	0
SCENT	4	TRL 8: Manufacturing, Ph 3 Clinical Trials, and FDA Approval	0
Other	1	Unspecified	10
**Total**	**49**	**Total**	**39**
			
			
**Institutional Background (N=39)**	**Count**	**Test Method (N=39)**	**Count**
Academic	23	Nucleic Acid Amplification Technology (NAAT), e.g., PCR	4
Private	6	Isothermal Nucleic Acid Amplification, e.g., LAMP, RPA	2
Undisclosed	10	Antigen detection, e.g., ELISA, EIA, LFA	4
**Total**	**39**	Direct Nucleic Acid Detection	2
		Direct Viral Secretion	1
		Undisclosed	6
**Test Characteristics**	**Count**	Other*	20
**(N=39, n=64)**		**Total**	**39**
Saliva	17		
Nasal Swab	11		
Aerosol/Breath	9	Method-Other*	**Count**
Oral Swab	6	Physical Signs	1
Blood	5	Immunological Markers	3
Wastewater	4	Chromatography	3
Other	4	Volatile Biomarkers	5
Sputum	3	Misc.	8
Olfactory or Other Sensory Function	3	**Total**	**20**
Fecal	1		
Secretions (e.g., sweat)	1		
**Total**	**64**		

Several key observations emerged from the survey results. First, many awardees came from academic institutions, which are traditionally research-focused and often lack experience in commercial product development. Second, most awardees were in the mid-stages of the TRL spectrum. While they had scientific expertise and a hypothesis for their diagnostic product, the majority were either still identifying a viable product candidate (6 groups), optimizing their prototype (12 groups), or in the early phases of manufacturing (7 groups).

Additionally, a broad range of testing methods were employed. While approximately one-third used traditional approaches such as nucleic acid amplification or antigen detection, the majority (20 out of 39 groups) selected “other” on the survey and provided a written response. We categorized these under the “Method-Other” classification, but some were so novel that they were grouped under “Miscellaneous” due to their unique approaches. This aligns with the core mission of RADx-rad, which was established to support exploratory and innovative diagnostic test development.

### B. Understanding the User Journey

**Fig. 1.**
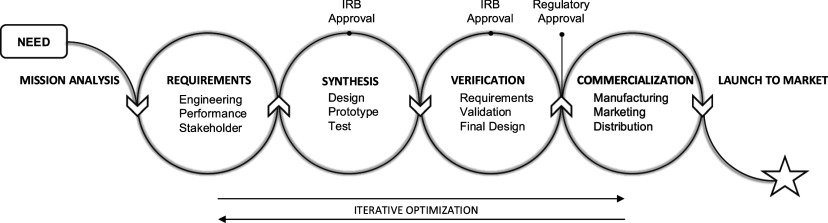
Diagnostic Innovation Journey Map. This map outlines the typical process that an awardee undergoes during the development of a diagnostic test. The journey begins with the identification of a target or need, followed by a mission analysis to determine the scope and feasibility of the project. The innovator then embarks on an iterative process that involves assessing the specific requirements for the diagnostic, synthesizing the technology, and verification of the prototype. Once these stages are successfully completed, the prototype progresses into the commercialization phase. The final step is the launch of the diagnostic product into the market.

The next step was understanding an awardee's typical journey in developing a diagnostic test. We drew inspiration from systems engineering principles [29], [30] but tailored it to the unique requirements of developing diagnostic medical technologies. We have outlined this as the Diagnostic Innovation Journey Map, illustrated in Fig. 1.

It begins with identifying a target or need, followed by a mission analysis to assess the diagnostic's objectives. The journey then moves into an iterative process of determining requirements, synthesizing the technology, and verifying the prototype. Once these stages are completed, the prototype advances to commercialization, with the final step being the launch of the diagnostic product into the market.

### C. Identifying Key Checkpoint

**Fig. 2.**
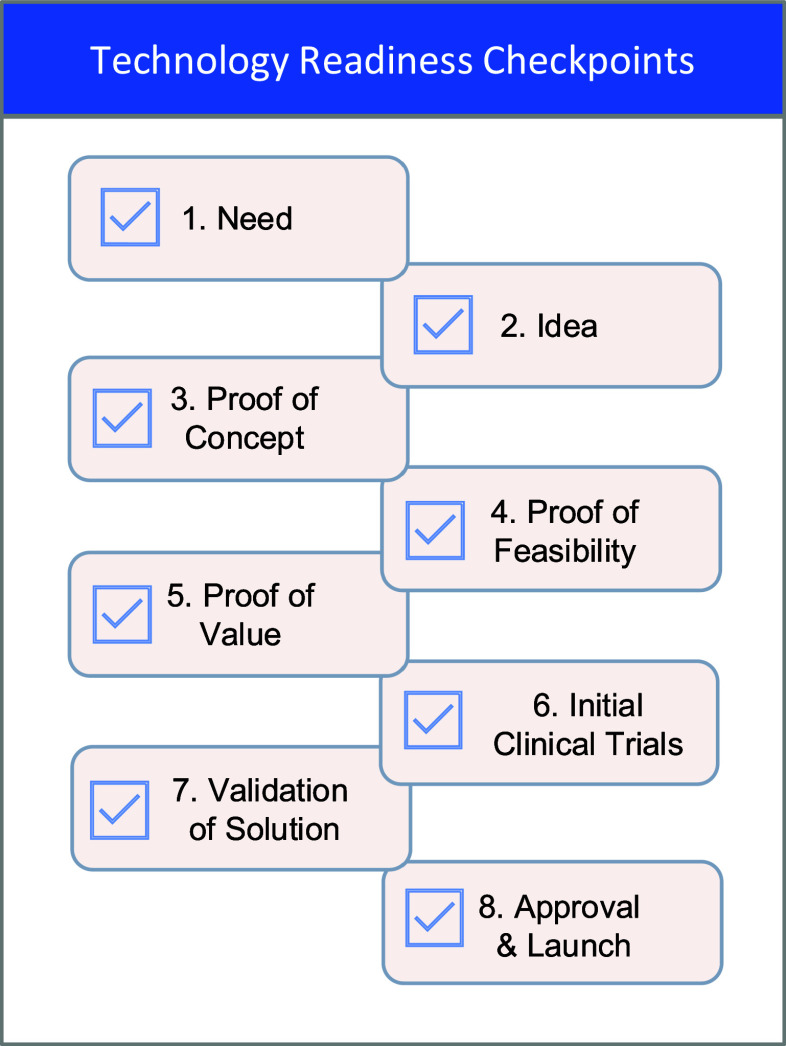
Diagnostic Technology Checkpoints. These are the critical checkpoints in the development of a diagnostic test. They include: 1. Identification of a need, 2. Formulation of a solution idea, 3. Demonstration of technology proof of concept, 4. Proof of feasibility, 5. Evidence of value, 6. Conducting initial clinical trials, 7. Validation of the solution, and finally 8. Launching the product to market.

Several key checkpoints must be achieved to develop and implement a new diagnostic technology successfully. Fig. 2 outlines eight critical milestones, adapted from the Technology Readiness Levels (TRLs) established by the Department of Defense (DoD) [31] and the CIMIT Healthcare Innovation Cycle [32]. The process begins with identifying a specific diagnostic need and then formulating an idea. After establishing a proof of concept, the next step is demonstrating feasibility under real-world conditions. The solution must then be validated for its value to key stakeholders, ensuring alignment with clinical and market requirements. Initial clinical trials assess safety, efficacy, and performance before advancing to larger-scale validation studies. The final stage involves securing regulatory approvals and launching the product for widespread adoption and use.

### D. Identifying Key Service Needs

**Fig. 3.**
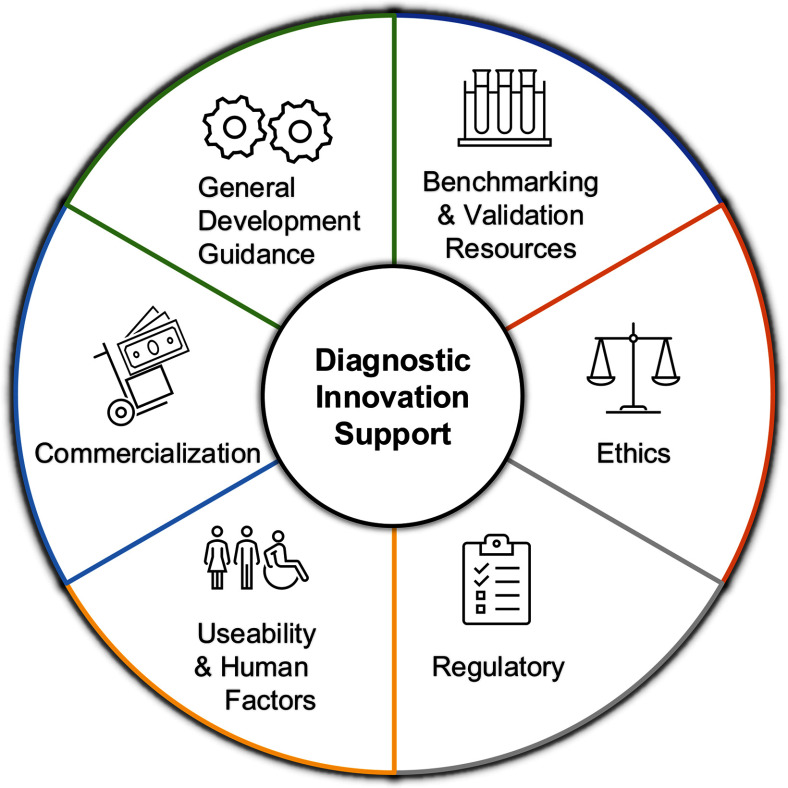
Key Components to Diagnostic Innovation Support. The critical components involved in supporting diagnostic innovation. These include providing resources for benchmarking and validation, general support for diagnostic development, ethical considerations, regulatory compliance, usability & human factors, and commercialization consulting.

This analysis identified six core services that would be most beneficial in supporting awardees throughout the diagnostic innovation process (Fig. 3). These services became the primary focus of the Dx Core program and include: (1) general guidance on diagnostic development, ensuring awardees receive expert support throughout the entire innovation process; (2) benchmarking and validation resources, providing access to testing samples and guidance on data analysis; (3) guidance on ethics, particularly in navigating the Institutional Review Board (IRB) process; (4) regulatory support, more specifically related to FDA processes; (5) usability and human factors guidance; and (6) strategic advice and support for commercialization. These focus areas formed the foundation of the Dx Core's service offerings, ensuring awardees received comprehensive support at every stage of diagnostic development.

### E. Develop a Service Model

**Fig. 4.**
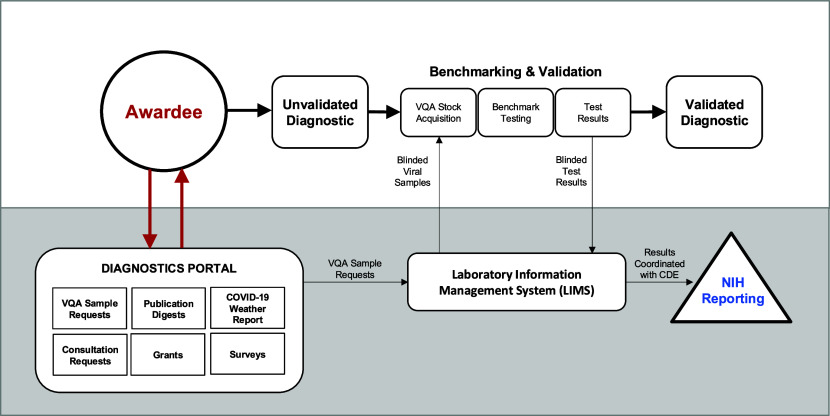
RADx-rad Diagnostic Core Service Model. Awardees access resources through the Dx Core Diagnostics Portal, an online platform to provide a variety of resources, including access to VQA sample request, publication digests, COVID-19 weather reports, consultation requests, grants, and surveys. When an awardee is ready to validate their diagnostic tests, VQA sample requests are sent to the LIMS system. The awardee is sent blinded viral samples on which to perform their benchmarking and validation testing. The results are submitted back to LIMS for unblinding. The unblinded results are then seamlessly integrated with the NIH Common Data Elements (CDE) system and reported to the NIH.

After defining the core services provided by the Dx Core, we developed a user-centered model to effectively deliver these resources to awardees (Fig. 4). The Diagnostic Portal, an online platform, served as the primary access point for these resources. Through the portal, awardees could request Viral Quality Assurance (VQA) samples, access publication digests, and COVID-19 weather reports, submit consultation requests, apply for grants, and complete surveys, ensuring streamlined and efficient support throughout the diagnostic development process.

A key objective of the Dx Core was to harmonize data across awardee groups and standardize reporting to the NIH. This was accomplished by providing standardized VQA samples, assisting with data analysis, and managing data submissions.

When an awardee's diagnostic test was ready for benchmarking and validation, they would submit a VQA sample request through the Diagnostic Portal to our Laboratory Information Management System (LIMS), which oversaw the VQA process. Standardized, blinded viral samples were sent to awardees for benchmarking and validation testing. After the testing, awardees submitted their blinded results back to LIMS, where they were unblinded and analyzed to generate standardized results, such as Receiver Operating Characteristic (ROC) curves and Limit of Detection (LOD) calculations [33]. These VQA samples were pre-coded with the NIH's Common Data Elements (CDEs), enabling automated, standardized data uploads to the NIH Data Hub.

This process upheld a high and consistent standard for diagnostic validation, ensuring reliable and comparable results across various tests. By offering standardized testing samples, ensuring consistent calculation of results, integrating NIH Common Data Elements, and managing data uploads, we provided a valuable service to both the NIH and the program awardees.

## III. Implementation

**Fig. 5.**
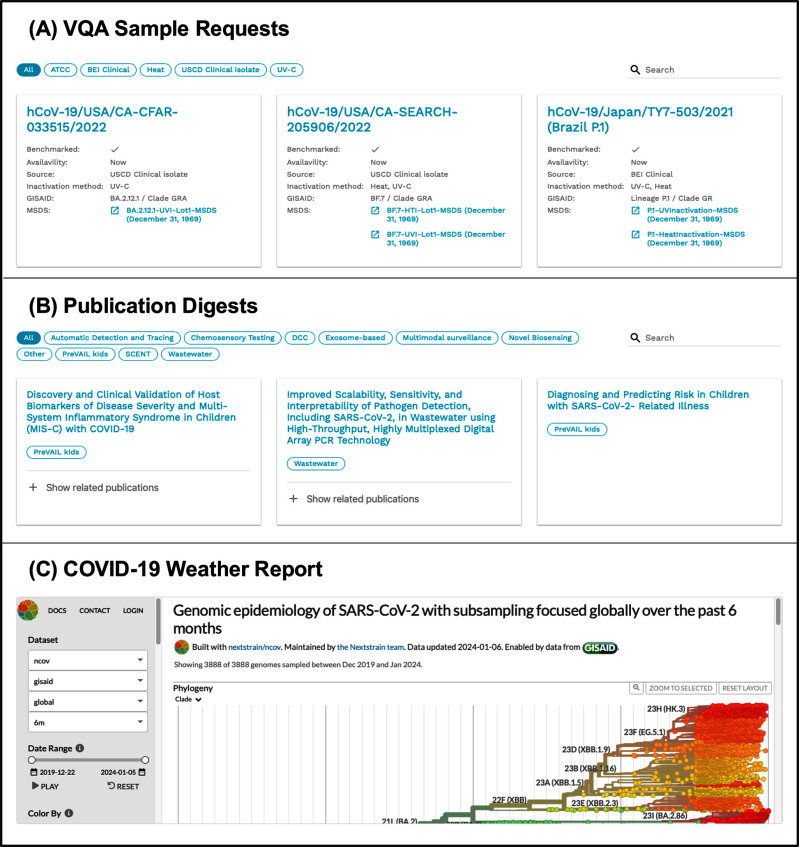
RADx-rad Diagnostic (Dx) Portal. These are snapshots from the Dx Core Web Portal, which was the primary point of interaction between the users and the Dx Core. The primary services offered were: (A) VQA Samples requests: This page allows users to view and request viral quality assurance (VQA) samples. Each listing provides detailed information on the viral strain's lineage, availability, and relevant metadata. (B) Publication digests: The publication digest feature offers curated articles, such FDA guidelines, emerging variants, and surveillance updates. Users can sign up for monthly email digests. (C) COVID-19 Weather report: The portal features a COVID-19 Weather report, a real-time tracking tool for pathogen evolution. It includes phylogenetic trees, transmission maps, and genome diversity visualizations, providing users with up-to-date insights on viral mutations and spread patterns.

The Dx Core operated over four years, December 2020 – December 2024, with the primary program development occurring within the first year. As mentioned, the Diagnostic Portal was the central hub for accessing key services. Fig. 5 provides sample images from the Diagnostic Portal (https://radxlab.org/) [34], developed by InSTEDD [35]. This online platform streamlined awardees’ access to essential resources and services.

The VQA Sample Request page (Fig. 5(A)) lists available viral samples, including the sample's origin, inactivation method (UV or heat), and links to relevant publications on the sample's lineage. The Publication Digest page (Fig. 5(B)) provided a searchable repository of SARS-CoV-2 diagnostic publications, covering topics such as new FDA approval guidelines, emerging variants evading current testing technologies, and wastewater surveillance updates. Users could also subscribe to receive monthly digests tailored to their areas of interest. The Weather Reports page (Fig. 5(C)) featured real-time pathogen evolution tracking powered by NextStrain [36], [37] and Rosalind Inc. [38], [39]. This tool included phylogenetic trees, transmission maps, and genome diversity visualizations. Based on user feedback, we expanded the tracker's scope beyond SARS-CoV-2 to cover other circulating viruses such as Influenza, RSV, and Mpox, making it a more comprehensive tool for monitoring emerging threats.

**Fig. 6.**
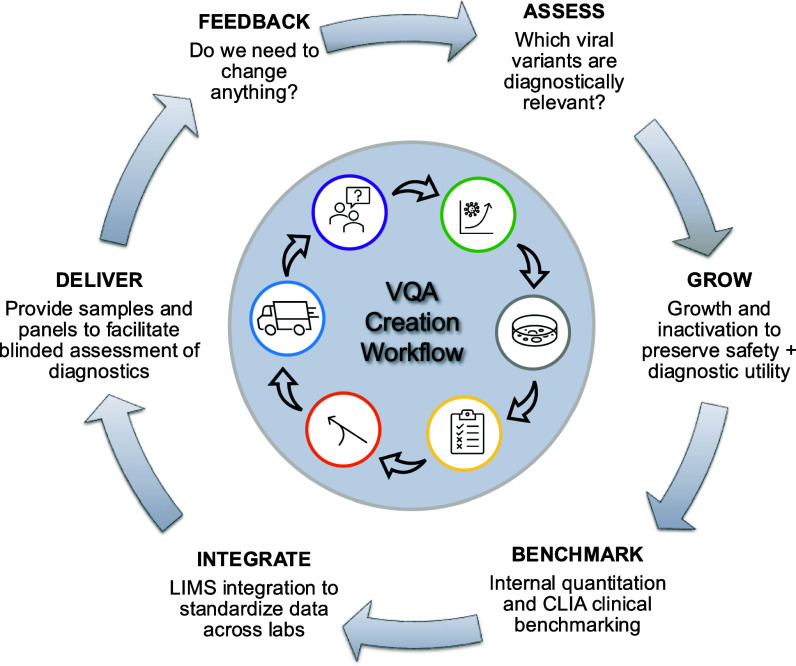
VQA Creation Workflow. VQAs are constantly updated to maintain stocks that are diagnostically relevant. The process followed to maintain stocks is (1) to assess which variants are diagnostically relevant, (2) grow and inactive those viral strains, (3) perform internal quantification and benchmarking, (4) integrate the sample data into LIMS, (5) provide samples and panels in a blinded format to awardees, and (6) received feedback and improve.

One of the most dynamic aspects of our services was the VQA sample stocks, which required continuous adaptation. As SARS-CoV-2 evolved, keeping our VQA sample stocks updated was crucial to ensure that awardees' diagnostics remained relevant to circulating strains. Fig. 6 outlines the continuous process used to update the Viral Quality Assurance (VQA) samples. This process included regularly evaluating the diagnostic relevance of viral variants, growing and inactivating these viruses, benchmarking the samples, and integrating them into the Laboratory Information Management System (LIMS) before distribution. Ongoing feedback from awardees was actively sought and used to make improvements as needed. As a result, awardees consistently had access to the most current and diagnostically relevant resources.

A more detailed schematic of the Dx Core's internal organization can be seen in **Supplementary Figure 1**.

## IV. Performance Outcomes

The Dx Core's performance was systematically evaluated over four years of operation, tracking key metrics across multiple domains, including general diagnostic development benchmarking and validation, ethical consultations, regulatory support, usability, commercialization, and grant funding. These efforts were assessed using internal tracking systems and periodic awardee surveys, and the results are summarized in Table II as key performance indicators (KPIs).

**TABLE II:** Key Performance Indicators (KPI). The KPIs Cover Benchmarking and Validation Resources, Consulting on Diagnostic Development, Ethics (IRB), Regulatory Compliance, Usability, Commercialization, and Access to Supplementary Grants

General Development Guidance			
# Office Hours	35	**Ethics**	
Digest Subscriptions	16	# Office Hours	30
D_x_ Core Seminars	4	# Individual Consultations	11
			
			
**Benchmarking & Validation Resources**		**Regulatory Support**	
# Samples Sent to Awardees	1333	# Awardee FDA meetings	28
# Awardees Groups Provided Samples	31	Total # Pre-EUAs submitted	7
VQA Sample Panels	Individual Samples	Total # EUAs submitted	1
	LOD Panel		
	Variant Panel		
	Challenge Panel	**Usability**	
Viruses Available	WA1	# Requests for Usability Resources	11
	Alpha B.1.1.7	# Requests for D_x_ Core consults	8
	Beta B.1.351		
	Gamma P.1		
	Delta B.1.617.2	**Commercialization Support**	
	Omicron BA.1	# SEED Meetings Granted	24
	Omicron BA.2.12.1	RADx-rad to RADx Tech	23
	Omicron BA.4.1	# Patents	32
	Omicron BA.5.1	# Licensing Agreements	10
	Omicron BF.7	# Awardee Publications	31
	Omicron BQ.1		
	Omicron XBB.1.5		
	CoV-229E	**Grants**	
	CoV-OC43	Supplemental Funding Awarded	#########
	RSV A	# Groups Awarded Supplemental Funding	10
	RSV B		
	Influenza A (H3N2)		
	Influenza A (H1N1)		
	Influenza B (2009)		

The outcomes of the Dx Core program demonstrate its success through the direct provision of resources and services, and indirectly through the awardees' achievements. One significant accomplishment was distributing 1333 viral samples for test validation. These samples were offered in various Viral Quality Assurance (VQA) panel formats designed to meet distinct validation needs. These included individual samples, Limit of Detection (LOD) panels for sensitivity analysis, Variant Panels to assess test performance across different SARS-CoV-2 strains, and Challenge Panels to evaluate specificity by distinguishing SARS-CoV-2 from other viruses.

Alongside providing physical resources, the Dx Core offered expert guidance through office hours, individual consultations, and specialized seminars covering essential topics such as diagnostic development, Institutional Review Board (IRB) ethics, and FDA regulatory approvals. The program also provided awardees with curated publications relevant to their focus areas through the Diagnostic Portal webpage and digest subscription.

Beyond the direct allocation of resources, the Dx Core's impact is also reflected in the success of the awardees themselves. Many made significant advancements in their technologies, as demonstrated by seven FDA pre-submissions, one full Emergency Use Authorization (EUA) submission, 32 patents, 10 licensing agreements, and 31 publications.

A key objective of the Dx Core was to facilitate the transition from early-stage development to commercialization. To accomplish this, the program partnered with the NIH Small Business Education and Entrepreneurial Development (SEED) Office [40], which offers entrepreneurial guidance and funding, and with RADx Tech [41], [42], [43], which supports the manufacturing and implementation of more mature technologies. While RADx-rad focused on early-stage and non-traditional diagnostics, RADx Tech was designed to support more commercially advanced technologies. A key milestone demonstrating the Dx Core's success was the transition of nearly half (23 of 49) of the awardees to the RADx Tech commercialization program.

Recognizing the financial challenges of product development, especially for awardees new to the field, the Dx Core also offered access to supplemental grants. More than $4 million dollars were allocated to assist awardees in advancing their diagnostic technologies.

The Dx Core's structured, user-centered approach provided awardees with access to essential resources, guidance and support throughout their diagnostic development journey. These outcomes reinforce the program's role in accelerating diagnostic innovation, enhancing pandemic readiness, and laying the groundwork for future public health response frameworks.

## V. Conclusion

The COVID-19 pandemic created an urgent need for the rapid development of diagnostic tests for SARS-CoV-2. To address this challenge, the NIH RADx-rad Dx Core was established to support NIH awardees in developing innovative, state-of-the-art diagnostic technologies. The Dx Core had two primary objectives: (1) to harmonize diagnostic test results and streamline NIH data submission, and (2) to provide essential resources to support the development of diagnostic tests. To achieve these goals, the Dx Core employed a strategic user-centered service model, the approach and outcomes of which are detailed in this paper.

The Dx Core's approach was grounded in three core principles: service design, convergence science, and quality control. Service design emphasizes the importance of building systems around the needs, experiences, and outcomes of the end-user—in this case, early-stage diagnostic innovators [44], [45], [46], [47], [48], [49]. Convergence science calls for collaboration across traditionally siloed disciplines to solve complex problems [51], [52]. The Dx Core operationalized this by integrating expertise from medicine, molecular biology, engineering, data science, clinical research, regulatory affairs, and product development to form a coordinated support system. Finally, quality control contributed a rigorously structured internal framework to ensure measurable outcomes and reproducible support processes [53], [54], [55].

This model proved highly effective. The Dx Core demonstrated how federal funding can be used efficiently and effectively to address an urgent public health need. One of the most impactful services was the distribution of 1333 viral samples in the form of standardized Viral Quality Assurance (VQA) panels, which helped ensure consistent performance validation across awardees’ tests. Additional services included expert-led office hours and seminars, curated publication digests, and real-time COVID-19 variant tracking, all accessible through a centralized online portal. These efforts equipped researchers with the tools, knowledge, and guidance necessary to move their technologies forward while also reducing the burden of regulatory and reporting requirements.

The program's success was also evident in awardee outcomes: 32 patents filed, 10 licensing agreements executed, 31 scientific publications, 7 FDA pre-submissions, and 1 full EUA submission. Perhaps most notably, 23 of the 49 awardee groups successfully advanced from early development to the RADx Tech commercialization pipeline. These achievements reflect the program's strong translational impact and illustrate how targeted support can accelerate the journey from concept to commercialization.

While a traditional return-on-investment (ROI) calculation may be difficult to quantify for a program of this nature, the tangible milestones achieved—scientific, regulatory, and commercial—demonstrate a high value for the investment. The Dx Core significantly accelerated diagnostic innovation at a time when speed and scalability were critical to national health security.

Beyond COVID-19, the Dx Core model serves as a scalable and adaptable framework for supporting diagnostic development in future public health emergencies. The infrastructure and workflows developed during this program can be applied to a range of other infectious diseases, including influenza, RSV, and emerging zoonotic threats. This foundation also supports the rapid deployment of resources in future pandemics, especially for early-stage developers who may lack access to essential tools and resources.

In conclusion, the RADx-rad Dx Core exemplifies how federal investment in a well-structured, interdisciplinary, and user-focused support system can catalyze innovation and enhance public health preparedness. By transforming a fragmented early-development landscape into a coordinated, high-functioning pipeline, the Dx Core improved both the quality and efficiency of diagnostic development. As new global health challenges emerge, this model stands as both a case study and a strategic framework for the future of diagnostic innovation and public health response.



Melissa Ledgerwood-Lee
UC San Diego Health
Department of Medicine
Division of Infectious Diseases
La Jolla, CA 92093, USA


Alexandra Hubenko
Qualcomm Institute
UC San Diego
La Jolla, CA 92093 USA

Partha Ray
UC San Diego Health
Department of Medicine
Division of Infectious Diseases
La Jolla, CA 92093, USA

Yves Theriault
UC San Diego Health
Department of Medicine
Division of Infectious Diseases
La Jolla, CA 92093, USA

Howard Brickner
UC San Diego Health
Department of Medicine
Division of Infectious Diseases
La Jolla, CA 92093, USA

Lidia F. Vazquez
UC San Diego Health
Department of Medicine
Division of Infectious Diseases
La Jolla, CA 92093, USA

Robert Schooley
UC San Diego Health
Department of Medicine
University of California San Diego
San Diego, CA 92130 USA

Aaron Carlin
UC San Diego Health
Department of Medicine
University of California San Diego
San Diego, CA 92130 USA

Alex Clark
UC San Diego Health
Department of Medicine
University of California San Diego
San Diego, CA 92130 USA

Aaron Garretson
UC San Diego Health
Department of Medicine
University of California San Diego
San Diego, CA 92130 USA

Eliah Aronoff-Spencer
UC San Diego Health
Department of Medicine
University of California San Diego
San Diego, CA 92130 USA


